# Editorial: Advancements in AI for the analysis and interpretation of large-scale data by omics techniques

**DOI:** 10.3389/fgene.2026.1784820

**Published:** 2026-02-13

**Authors:** Angelo Facchiano, Deborah Giordano, Dominik Heider

**Affiliations:** 1 National Research Council, Institute of Food Science, Avellino, Italy; 2 Institute of Medical Informatics, University of Münster, Münster, Germany

**Keywords:** artificial intelligence, bioinformatics, complex biological data, human pathologies, OMICS techniques

The rapid expansion of omics technologies has generated unprecedented volumes of complex biological data, creating both new opportunities and major analytical challenges in bioinformatics. In this context, artificial intelligence has emerged as a powerful approach to enhance data integration, interpretation, and biological insight. The Research Topic “*Advancements in AI for the Analysis and Interpretation of Large-scale Data by Omics Techniques*” has been conceived as a collection of articles bringing together recent advances, methodologies, and perspectives on the application of AI to omics data analysis, highlighting innovative tools, real-world applications, and future directions. By bridging computational innovation and biological discovery, the contributions in this Research Topic aim to advance our understanding of molecular systems and to foster more robust, interpretable, and scalable solutions in modern bioinformatics. The articles that passed the selection process, conducted according to the journal’s standard peer-review procedures, are all directly focused on human pathology, confirming a strong interest in the application of AI techniques in the medical field.

The Research Topic includes seven contributions: in detail one “Opinion” article and six “Original Research” articles.

The Opinion article by Ribeiro et al., entitled “*From bites to bytes: understanding how and why individual malaria risk varies using artificial intelligence and causal inference*”, concerns the advancements in the study of malaria, a major global health challenge, driven by complex interactions among biological, environmental, behavioral, and socioeconomic factors. The article discusses how integrating Artificial Intelligence, Machine Learning, and causal inference can improve malaria research by identifying context-specific risk factors and uncovering causal mechanisms. Using longitudinal, multimodal data from the Mâncio Lima cohort and related Amazonian studies, it shows how AI-driven and privacy-preserving approaches, including federated learning, enable refined risk stratification and actionable insights, supporting precision public health strategies for more equitable and effective malaria control.

The original research article entitled “*Single-cell analysis and machine learning identify psoriasis-associated CD8* ^*+*^ *T cells serve as biomarker for psoriasis*”, by He et al., employs single-cell techniques to analyze psoriasis, a chronic inflammatory skin disease characterized by complex immune mechanisms in which CD8^+^ T cells play a key pathogenic role. The study investigates psoriasis at single-cell resolution, identifying a distinct CD8^+^ T cell subpopulation highly enriched in psoriatic lesions. Using hdWGCNA, key hub genes were characterized, and a machine-learning–based predictive model with strong performance was developed and interpreted. To support clinical translation, the model was deployed as an online tool, offering new insights for diagnosis and potential therapeutic targeting.

In the original research article “*AnchorFCI: harnessing genetic anchors for enhanced causal discovery of cardiometabolic disease pathways*,” Ribeiro et al., present a novel causal discovery method named anchorFCI that enhances robustness and discovery power by integrating reliable genetic anchor variables. Through simulations and analysis of the 2015 ISA-Nutrition dataset, anchorFCI supports known causal relationships while revealing new interconnections among cardiometabolic risk factors. Combined with state-of-the-art effect identification tools, this approach provides a robust, data-driven framework for causal inference in complex epidemiological and public health studies.

Hu and Fang, the authors of the original research article entitled “*Explore potential immune-related targets of leeches in the treatment of type 2 diabetes based on network pharmacology and machine learning*,” investigate the potential mechanisms of leeches, a traditional Chinese medicine, in type 2 diabetes treatment using network pharmacology, transcriptomics, and machine-learning approaches. By integrating database mining, enrichment analyses, immune infiltration profiling, WGCNA, and multiple predictive algorithms, the study identifies potential therapeutic targets linked to immune modulation. Although promising, these findings require further experimental validation to confirm their clinical relevance.

The original research article entitled “*Prediction of mild cognitive impairment using blood multi-omics data*,” by Zhang et al., concerns mild cognitive impairment (MCI), an early stage of cognitive decline and a key risk factor for Alzheimer’s disease, which is still difficult to diagnose. This study presents a blood-based, multi-omics machine-learning approach for MCI detection, integrating gene expression and copy number variation data. An XGBoost model achieved high predictive performance (AUC = 0.9398), demonstrating for the first time that genome-structure–level features are as informative as gene expression. Key genomic predictors were enriched in neurodegeneration-related pathways, highlighting both the diagnostic potential and biological relevance of this approach.

In the original research article entitled “*Reference-free deconvolution of complex samples based on cross-cell-type differential analysis: Systematic evaluations with various feature selection options*,” Zhang et al. present a novel reference-free deconvolution method that leverages optimized feature selection through cross–cell-type differential analysis. By systematically evaluating feature selection strategies and iteratively identifying cell-type-specific features, the proposed approach achieves high accuracy. Extensive simulations and analyses of multiple real datasets demonstrate its strong performance.

Celli et al., in the original research article entitled “*scVAR: integrating genomics and transcriptomics from single-cell RNA-seq—insights from leukemia case studies*,” present a computational framework that integrates genetic variation and transcriptomic information directly from single-cell RNA sequencing data. This powerful and broadly applicable approach enables integrative single-cell analysis of complex diseases. Using a variational autoencoder architecture with cross-attention–based fusion, the framework captures subtle cellular heterogeneity under noisy and sparse conditions. Applied to acute leukemias, the method identifies substantially more cellular subpopulations than transcriptomic analysis alone, revealing cell identities otherwise overlooked.

Overall, this Research Topic has attracted considerable interest within the scientific community and brings together a series of high-quality, forward-looking contributions that highlight the transformative potential of artificial intelligence in omics research. Taken together, these articles not only address current methodological and biomedical challenges but also open new perspectives for future research, emphasizing the growing role of artificial intelligence-based approaches in shaping the next-generation of precision medicine and biomedical discoveries.

